# Molecular markers of resistance to amodiaquine plus sulfadoxine–pyrimethamine in an area with seasonal malaria chemoprevention in south central Niger

**DOI:** 10.1186/s12936-018-2242-4

**Published:** 2018-02-27

**Authors:** Rebecca F. Grais, Ibrahim M. Laminou, Lynda Woi-Messe, Rockyath Makarimi, Seidou H. Bouriema, Celine Langendorf, Alfred Amambua-Ngwa, Umberto D’Alessandro, Philippe J. Guérin, Thierry Fandeur, Carol H. Sibley

**Affiliations:** 1 Epicentre, Paris, France; 2 CERMES, BP 11887, Niamey, Niger; 3 Epicentre Niger, BP 13330, Niamey, Niger; 4 PNLP, BP 10514, Niamey, Niger; 5 MRC Gambia, Serrekunda, The Gambia; 6 WorldWide Antimalarial Resistance Network, University of Oxford, Oxford, UK; 7 Division Internationale, Institut Pasteur, 28 rue du Dr Roux, 75725 Paris Cedex 15, France; 8 WorldWide Antimalarial Resistance Network, University of Washington, Seattle, USA

**Keywords:** Malaria, Seasonal malaria chemoprophylaxis, Niger, Prevention

## Abstract

**Background:**

In Niger, malaria transmission is markedly seasonal with most of the disease burden occurring in children during the rainy season. Seasonal malaria chemoprevention (SMC) with amodiaquine plus sulfadoxine–pyrimethamine (AQ + SP) is recommended in the country to be administered monthly just before and during the rainy season. Moreover, clinical decisions on use of SP for intermittent preventive treatment in pregnancy (IPTp) now depend upon the validated molecular markers for SP resistance in *Plasmodium falciparum* observed in the local parasite population. However, little is known about molecular markers of resistance for either SP or AQ in the south of Niger. To address this question, clinical samples which met clinical and biological criteria, were collected in Gabi, Madarounfa district, Maradi region, Niger in 2011–2012 (before SMC implementation). Molecular markers of resistance to pyrimethamine (*pfdhfr*), sulfadoxine (*pfdhps)* and amodiaquine (*pfmdr1)* were assessed by DNA sequencing.

**Results:**

Prior to SMC implementation, the samples showed a high proportion of clinical samples that carried the *pfdhfr* 51**I**/59**R**/108**N** haplotype associated with resistance to pyrimethamine and *pfdhps* 436**A/F/H** and 437**G** mutations associated with reduced susceptibility to sulfadoxine. In contrast mutations in codons 581**G**, and 613**S** in the *pfdhps* gene, and in *pfmdr1,* 86**Y**, 184**Y**, 1042D and 1246**Y** associated with resistance to amodiaquine, were less frequently observed. Importantly, *pfdhfr* I164**L** and *pfdhps* K540**E** mutations shown to be the most clinically relevant markers for high level clinical resistance to SP were not detected in Gabi.

**Conclusions:**

Although parasites with genotypes associated with the highest levels of resistance to AQ + SP are not yet common in this setting, their importance for deployment of SMC and IPTp dictates that monitoring of these markers of resistance should accompany these interventions. This study also highlights the parasite heterogeneity within a small spatial area and the need to use caution when extrapolating results from surveys of molecular markers of resistance in a single site to inform regional policy decisions.

## Background

Although there has been great progress in malaria control, fewer than half of endemic countries account for 85% of all reported cases and most of these are in Africa [1]. In Niger, according to official estimates, the total number of confirmed and presumptive malaria cases was 846,509 in 2012 which roughly corresponds to a malaria incidence rate of 263 per thousand and malaria-related mortality was 0.12% [2]. While the entire population is exposed to the risk of malaria, the most vulnerable groups are pregnant women and children under the age of 5 years [3, 4]. Niger is within the Sahel, where malaria is markedly seasonal and most disease occurs during the rainy season from June to September. The epidemiological situation in Niger is thus suitable for the implementation of seasonal malaria chemoprevention (SMC) in children. This treatment involves the monthly administration of sulfadoxine–pyrimethamine (SP) and amodiaquine (AQ) during the season of high transmission when malaria endemicity and mortality are the highest [4]. Several studies have shown that SMC provides protection against malaria, resulting in a decrease in the number of clinical episodes and mortality, and in the incidence of severe malaria, but logistical challenges are formidable in some parts of eligible regions [5–8].

Malaria episodes during pregnancy compromise the health of the mother and the development of her fetus, particularly in primiparous women [9]. The World Health Organization (WHO) recommends that in high transmission areas, SP be given at least a month apart at the 4 antenatal visits after the first trimester for preventive treatment of malaria (IPTp) [10]. In many regions, clinical responses to SP are seriously compromised, and SP is no longer recommended for treatment of malaria episodes. However, it has been difficult to determine whether the efficacy of SP for IPTp is similarly compromised.

These uncertainties mean that decisions about whether to introduce SMC or IPTp as additional malaria preventive strategies are partly dependent on the local level of resistance to the antimalarial drugs being used [9–11]. Epidemiological monitoring of susceptibility to SP can be estimated by measuring the prevalence in the parasite population of key mutations that serve as surrogate molecular markers of the clinical response to anti-malarials. For SP, the prevalence of parasites that carry the “quintuple mutant” genotype, both the triple mutant haplotype, *pfdhfr* allele N51**I**/C59**R**/S108 **N** and the double mutant haplotype, A437**G** and K540**E** mutations of the *pfdhps* gene are strongly associated with clinical failure of SP treatment [10, 11]. Similarly, parasites that carry a N86**Y**/F184**Y**/D1246**Y** haplotype of the *pfmdr1* gene are associated with reduced susceptibility to amodiaquine [12–15]. Whether SP for IPTp or SP + AQ for SMC is still protective in locations where these genotypes are common is under active investigation. Therefore, surveillance of these and other additional markers of SP and AQ response is a crucial element of future clinical decisions on implementation of SMC and IPTp in Niger.

Information on resistance to anti-malarials is scarce in Niger, but an evaluation of these markers of SP and AQ resistance can provide a baseline with which to assess any changes in these molecular markers associated with the implementation of SMC. To that end, this study reports the prevalence of mutations of the *pfdhfr*, *pfdhp*s and *pfmdr1* genes associated with resistance to SP and AQ in *Plasmodium falciparum* blood samples collected from children living in a semi-urban area of Niger before and during the implementation of SMC.

## Methods

### Study site

**Fig. 1:**
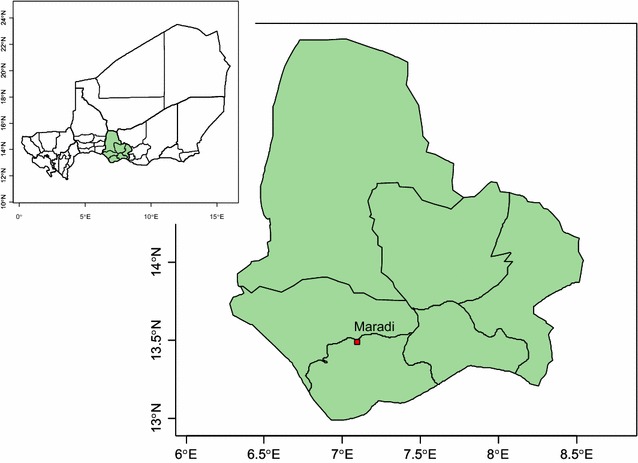
Map showing the location of the Maradi region in Niger and the location of the capitol, Maradi city where the Gabi Health Centre is located

The study was conducted in at the Gabi community health centre, Madarounfa district, Maradi Region along the southern border of Niger (Fig. 1). The rainy season extends from June to September with an annual average (2000–2012) precipitation of 445 mm. A previous study showed that the Maradi Region is meso-to hyperendemic for malaria with a rate of slide-positivity for inpatients peaking at 70% during the season of high transmission and falling to 20% during the dry season when the transmission rate is the lowest [16].

### Sample collection

Samples for molecular analysis were collected from young children presenting at the Gabi health centre. After clinical examination, children presenting with fever (axillary temperature ≥ 37.5 *°*C) or history of fever during the previous 24 h were tested for malaria. The presence of *P. falciparum* was initially investigated by a rapid diagnostic test (HRP2 SD Bioline^®^) then confirmed and quantified by microscopy on Giemsa-stained blood smears. Children with uncomplicated *P. falciparum* infection with parasite density between 2000 and 200,000 per microlitre, aged 6–59 months and/or height greater than 65 cm, and weighing more than 5 kg, were identified and their parents or caregivers invited to participate in the study. Exclusion criteria were the presence of mixed infections with species other than *P. falciparum* or presence of severe malaria or general danger signs; these children were immediately referred for inpatient evaluation. The recruitment period extended from December 2011 to January 2012. After obtaining informed consent from parents or guardians, 1 ml of venous blood was collected in EDTA anticoagulant tubes. Parasitized blood samples were stored frozen at – 20 °C until analysis.

### Ethical considerations

This study was authorized by the Ministry of Health of Niger, and ethical clearance was obtained from the National Ethical Committee of Niger (authorization N°022/2011/CCNE and N°003/2013/CCNE). Documents relating to the consent procedure were translated into Hausa which is the formal local language. The information sheet detailing the purpose of the study was read in its entirety to the parents or guardians, and a copy of the document was provided. An interview was given to every participant and to parents after which the consent form was signed, or validated by a fingerprint for illiterate persons. All subjects testing positive for malaria, included or not in the study, were treated without charge according national protocols. Data from this study have been deposited in the WorldWide Antimalarial Resistance Network repository for open use in individual patient data meta-analyses.

### DNA extraction and PCR

Genomic DNA was extracted according to manufacturer’s recommendations (QIAamp DNA blood kit, QIAGEN). Measurement of the concentration and purity of DNA was achieved using a NanoDrop2000 UV-Vis spectrophotometer. All amplifications were done in a microtube format, using a Primus 96 thermocycler (PEQLAB Biotechnology). To analyse parasite populations that were as homogeneous as possible, clinical samples were first typed by amplification of polymorphic block 2 sequences of *pfmsp2* essentially as described elsewhere [15]. Those producing a single fragment by agarose gel electrophoresis were judged to be monoclonal and selected for analysis of markers associated with resistance. *pfdhfr* and *pfdhps* sequences were amplified by nested PCR as described elsewhere [16]. Briefly, M1 (5′TTTATGATGGAACAAGTCTGC3′) and M7 (5′CTAGTATATACATCGCTAACA3′) oligonucleotides were used for primary amplification of *pfdhfr* sequences (650 bp) and M3b (5′TGATGGAACAAGTCTGCGACGTT3′) and M9 (5′CTGGAAAAAATACATCACA TTCATATG3′) for secondary reactions, producing a 594 bp fragment containing key mutations A16V/S, N51I, C59R, S108N, V140L and I164L. For *Pfdhps* sequences a 770 bp fragment was first amplified using N1 (5′GATTCTTTTTCAGATGGAGG3′) and N2 (5′TTCCTCATGTAATTCATCTG A3′) then used as template for secondary amplifications using primers R2 (5′AACCTAAACGTGCTGTTCAA3′) and R (5′AATTGTGTGA TTTGTCCACAA3′). The final PCR product (711 bp) contains mutations S436A/F, A437G, K540E, A581G, and A613S, previously associated with parasite responses to sulfonamide. Detailed PCR conditions for amplification of these markers are as described elsewhere [17]. Primers for cycle-sequencing included; Pfdhps_Fwd (AACCTAAACGTGCTGTTCAA), Pfdhps_Rev (AATTGTGTGATTTGTCCACAA), Pfdhps_IntFwd1 (ATTCTATAGTGTAGTTCTAATGC), Pfdhps_IntRev2 (CTGGATTATTTGTACAAGCAC), Pfdhfr_Fwd (TGATGGAACAAGTCTGCGACGTT), Pfdhfr_Rev (CTGGAAAAAATACATCACATTCATATG). Cycle sequencing follow standard BigDye3.1 dye terminator protocol (AppliedBiosystems) on an MJ-Thermocylcer. Sequencing reactions were cleaned on Sephadex G10 columns and analysed on ABI3130xl Genetic Analyser.

### Data analysis

The enrolment period was 1 month, in order to collect approximately 150 blood samples. This sample size allows reliable assessment of a prevalence of 12% (corresponding to the prevalence of parasites that carried the triple *pfdhdr* mutation previously observed in Niger [16]) with a precision of ± 7%. Data were double-entered by two independent entry clerks into EPIinfo version 3.5.2 (Odensk, Denmark). General characteristics of the population were described by mean, standard deviation and range for continuous variables and by absolute number and percentage for categorical variables.

## Results

A total of 201 blood samples were collected and processed for DNA amplification. The mean age and the mean body weight (and standard deviation) of the patients included in the study were 22.8 ± 11.7 months [range: 6–55] and 9.5 ± 5.7 kg [range: 5.3–15.5], respectively, with a sex ratio of 1.04 in favour of girls. Patients included in the study had a *P. falciparum* monoinfection with a median parasite density 14,076 parasites/mm [range 80–202,000]). Of the 201 samples amplified by PCR with *pfdhfr*, *pfdhps* and *pfmdr1* primers, 120 (59.7%), 103 (51.2%), and 95 (47.2%), respectively, produced fragments in sufficient yield for sequencing. The electropherograms showed that 13.8, 11.1 and 10.8% of the amplified *pfdhfr, pfdhps,* and *pfmdr1* sequences, respectively, were heterogeneous consisting of a mixture of wild type and mutated residues at one or more individual polymorphic positions. These polyclonal clinical samples were not included for the calculation of the allele prevalence and reconstruction of the various haplotypes. In total, 136 amplicons of *pfdhps* and 120 amplicons of *pfdhfr* were sequenced. Combined genotypes were established using the samples that were successfully analysed at one (p*fdhfr*, *pfdhps* or *pfmdr1*), two (*pfdhfr* and *pfdhps*), or three (*pfdhfr, pfdhps* and *pfmdr1*) loci.

### Prevalence of individual point mutations in *pfdhfr*, *pfdhps* and *pfmdr1* genes

Prevalence of individual mutant codons was determined in the apparently monoclonal clinical samples. These samples showed a high prevalence (> 60%) of mutations N51**I**, C59**R** and S108**N** in *pfdhfr*, known to be associated with resistance to pyrimethamine. The S108**N** mutation was the most represented with a prevalence of 69%, whereas the mutation 16**V/S**, associated with resistance to cycloguanil [18] and mutation I164**L**, previously described for some highly resistant parasites of Asian origin [19] and very rare African origins were not detected [12]. Five SNPs were genotyped in the *pfdhps* gene at codons 431, 436, 437, 540, 581 and 613, in the *pfdhps* locus; the key mutation at codon K540**E** was not observed, but all other mutations associated with modulation of the responses of parasites to sulfadoxine were detected at variable frequencies. The mutations in codons S436**A/F/G** and A437**G** were found in 65 and 83% of isolates, respectively, while A581G and A613**S** mutations were less frequent (0.1–0.25). Four point mutations associated with reduced responses to amodiaquine were detected in *pfmdr1* sequences in codons 86, 184, 1042 and 1246. The prevalence of a mutation changing codon F184**Y** was 37% whereas the N86**Y**, N1042**D** and D1246**Y** mutations were detected at very low prevalence.

### Prevalence of *pfdhfr*, *pfdhps* and *pfmdr1* haplotypes

Haplotypes were reconstituted by including all unambiguous data collected at the designated polymorphic positions in *pfdhfr*, *pfdhps* and *pfmdr1* sequences (Table 1). The wild type genotype N51/C59/S108 (NCS) was identified in 27% (32/118) of the isolates while the triple mutant 51**I**/59**R**/108**N** (**IRN**) haplotype was detected in 57% (67/118) of the evaluable clinical samples. The **I**CS and NC**N** haplotypes (single mutants) and **IR**S, N**RN** and **I**C**N** (double mutants) were less prevalent at 6% (7/118) and 11% (12/118), respectively. The *pfdhps* genotypes were defined according to residues found at positions 437, 540, 581 and 613. A total of 5 distinct genotypes were observed. Fourteen % (14/108) of the clinical samples carried only the wild type A437/K540/A581/A613 (AKAA) haplotype, single mutant haplotypes **G**KAA, AKA**S**, and AK**G**A, a double mutant, **G**KA**S**, and a triple mutant **G**A**GS** (triple mutant) represent 66% (71/108), 13.% (14/108) and 8% (9/108) of the clinical samples tested, respectively. Single and doubly mutated genotypes predominate in Niger and represent 78% of the clinical samples.

**Table 1:** Sequence polymorphisms in *pfdhfr*, *pfdhps* and *pfmdr1* genes

Marker	Category	Haplotype	Number	Prevalence
*pfdhfr 51,59,108*	Wild type	NCS	32	0.27
	Single mutant	**I**CS	3	0.03
		NC**N**	4	0.03
	Double mutant	**IR**S	1	0.01
		N**RN**	3	0.03
		**I**C**N**	8	0.07
	Triple Mutant	**IRN**	67	0.57
*pfdhps 437,540, 581, 613*	Wild type	AKAA	14	0.14
	Single mutant	**G**KAA	66	0.67
		AKA**S**	4	0.04
		AK**G**A	1	0.01
	Double mutant	**G**KA**S**	14	0.14
	Triple mutant	**G**K**GS**	9	0.08
*pfmdr1 86,184,1042,1246*	Wild type	NFND	63	0.52
	Single mutant	**Y**FND	2	0.02
		N**Y**ND	38	0.31
	Double mutant	N**FD**D	5	0.04
		N**F**N**Y**	3	0.03
		**YY**ND	5	0.04
		N**YD**D	1	0.01
		N**Y**N**Y**	1	0.01
		NF**DY**	1	0.01

The number and prevalence of the various haplotypes are indicated. Residues considered as mutated are underlined and in bold

The *pfmdr1* haplotypes were defined by identifying the residues at polymorphic codons 86, 184, 1042 and 1246. The wild-type sequence NFND was the most prevalent, 52% (63/121). Haplotypes carrying a single (**Y**FND, N**Y**ND, NF**D**D, NFN**Y**) or a double mutation (**YY**DD, N**YD**D, N**Y**N**Y**, NF**DY** and **Y**FN**Y**) were found in 40% (48/121) and 8% (10/121) of the clinical samples. No *pfmdr1* genotypes harbouring more than two individual mutated codons were detected.

### Multilocus *pfdhfr/pfdhps* and *pfdhfr/pfdhps*/*pfmdr1* genotypes

Thirty-six clinical samples had full sequence information for all three loci. Overall, these parasites carried a wide variety of mutations associated with resistance to SP and AQ; there was no isolate that was fully wild type at either *pfdhfr*/*pfdhps* or *pfdhfr*/*pfdhps*/*pfmdr*. With respect to genotypes associated with SP resistance, clinical samples that carried 3 mutations in the *pfdhfr* sequences and the single 437G in *pfdhps* (**IRN** + **G**KAA) constituted 26% (9/36)—of the clinical samples, but the quintuple mutant (**IRN** + **GE**AA) most commonly associated with clinical failure of SP treatment was not observed. However, four clinical samples (0.11) did carry a different quintuple mutant (**IRN** + **G**KA**S**) that may be implicated in SP resistance [17]. The analysis of multilocus genotypes at the *pfdhfr*/*pfdhps*/*pfmdr* loci showed that only two clinical samples carried both a triple mutant *pfdhfr* (**IRN**) and a 184**Y** allele at the *pfmdr1* locus; among these, only 1 also carried a double mutant *pfdhps* allele (**G**KA**S)** (Table 2).

**Table 2:** Detail and prevalence of multilocus genotypes in *P. faciparum* clinical samples from Gabi, Madarounfa, Maradi, Niger

*pfdhfr*			*pfdhps*					*pfmdr1*				Haplotypes		
N51*I*	C59*R*	S108*N*	I431*V*	S436*A/F*	A437*G*	A581*G*	A613*S*	N86*Y*	F184*Y*	N1042*D*	D1246*Y*	*dhfr/dhps/mdr1*	Number	Prevalence
*I*	C	S	I	S	*G*	A	A	N	*Y*	N	D	*I*CS/IS*G*AA/N*Y*ND	1	0.01
*I*	C	S	I	S	*G*	A	A	N	F	N	D	*I*CS/IS*G*AA/NFND	1	0.01
N	C	S	I	*A*	A	*G*	A	N	F	N	D	NCS/I*A*A*G*A/NFND	1	0.01
N	C	S	I	*A*	G	A	*S*	N	F	N	D	NCS/I*AG*A*S*/NFND	1	0.01
N	C	S	I	*A*	A	A	A	N	F	*D*	*Y*	NCS/I*A*AAA/NF*DY*	1	0.01
*I*	*R*	S	I	*A*	*G*	A	*S*	N	*Y*	N	D	*IR*S/I*AG*A*S*/N*Y*ND	1	0.01
*I*	*R*	*N*	I	S	*G*	A	A	N	*Y*	*D*	D	*IRN*/IS*G*AA/N*YD*D	1	0.01
N	*R*	*N*	I	*A*	*G*	A	A	N	F	N	D	N*RN*/I*AG*AA/NFND	1	0.01
*I*	*R*	*N*	I	*A*	A	A	A	N	F	N	D	*IRN*/I*A*AAA/NFND	1	0.01
*I*	*R*	S	I	*A*	*G*	A	*S*	N	F	N	D	*IR*S/I*AG*A*S*/NFND	1	0.01
*I*	C	*N*	I	*A*	*G*	A	A	N	F	N	D	*I*C*N*/I*AG*AA/NFND	1	0.01
*I*	C	*N*	*V*	*A*	*G*	A	A	N	F	N	D	*I*C*N*/*VAG*AA/NFND	1	0.01
*I*	*R*	*N*	I	*A*	A	A	A	N	F	*D*	D	*IRN*/I*A*AAA/NF*D*D	1	0.01
*I*	C	*N*	*V*	*A*	*G*	A	A	*Y*	F	N	D	*I*C*N*/*VAG*AA/*Y*FND	1	0.01
*I*	*R*	*N*	I	*A*	A	A	*S*	N	F	N	D	*IRN*/I*A*AA*S*/NFND	1	0.01
*I*	*R*	*N*	I	*F*	*G*	A	S	N	*Y*	N	D	*IRN*/I*FG*A*S*/N*Y*ND	1	0.01
N	C	*N*	*V*	*A*	*G*	A	A	N	F	N	*Y*	NC*N*/*VAG*AA/NFN*Y*	1	0.01
N	*R*	*N*	I	*A*	*G*	A	*S*	N	F	N	D	N*RN*/I*AG*A*S*/NFND	1	0.01
N	C	*N*	*V*	*A*	*G*	A	A	*Y*	F	N	*Y*	NC*N*/*VAG*AA/YFN*Y*	1	0.01
*I*	*R*	*N*	I	*F*	*G*	A	*S*	N	F	N	D	*IRN*/I*FG*A*S*/NFND	1	0.01
*I*	*R*	*N*	*V*	*A*	*G*	A	A	N	F	N	D	*IRN*/*VAG*AA/NFND	1	0.01
*I*	*R*	*N*	*V*	*A*	*G*	A	A	*Y*	F	N	D	*IRN*/*VAG*AA/YFND	1	0.01
*I*	*R*	*N*	*V*	*A*	*G*	A	A	*Y*	F	N	*Y*	*IRN*/*VAG*AA/YFNY	1	0.01
N	C	S	I	*A*	A	A	A	N	F	N	D	NCS/I*A*AAA/NFND	2	0.02
N	C	S	I	*A*	*G*	A	A	N	F	N	D	NCS/I*AG*AA/NFND	2	0.02
*I*	*R*	*N*	I	S	A	A	A	N	F	N	D	*IRN*/ISAAA/NFND	2	0.02
*I*	*R*	*N*	I	S	*G*	A	A	N	F	N	*Y*	*IRN*/IS*G*AA/NFN*Y*	2	0.02
*I*	*R*	*N*	I	S	A	A	A	N	*Y*	N	D	*IRN*/ISAAA/N*Y*ND	3	0.03
*I*	*R*	*N*	I	*F*	A	A	*S*	N	*Y*	N	D	*IRN*/I*F*AA*S*/NYND	3	0.03
*I*	*R*	*N*	I	*F*	A	A	*S*	N	F	N	D	*IRN*/I*F*AA*S*/NFND	3	0.03
*I*	*R*	*N*	I	*A*	*G*	A	*S*	N	F	N	D	*IRN*/I*AG*A*S*/NFND	3	0.03
N	C	S	I	*A*	A	A	A	N	F	*D*	D	NCS/I*A*AAA/NF*D*D	4	0.04
N	C	S	I	S	*G*	A	A	N	*Y*	N	D	NCS/IS*G*AA/N*Y*ND	7	0.08
*I*	*R*	*N*	I	S	*G*	A	A	N	*Y*	N	D	*IRN*/IS*G*AA/N*Y*ND	9	0.10
*I*	*R*	*N*	I	S	*G*	A	A	N	F	N	D	*IRN*/IS*G*AA/NFND	10	0.11
*I*	*R*	*N*	I	*A*	*G*	A	A	N	F	N	D	*IRN*/I*AG*AA/NFND	18	0.20

Mutated residues are in italic

## Discussion

Parasites carrying 51**I**/59**R**/108**N** triple-mutant allele of *pfdhfr* and the 437**G**/540**E** allele of *pfdhps* are major contributors to SP treatment failure [12, 20–22]. In most West African countries, the *pfdhps* allele with the single mutant, 437**G** are common, but parasites with the triple mutant *pfdhfr* and the key 437**G**/540**E** substitutions are still rare (see WWARN SP surveyor).

SP intermittent preventive treatment of women in the last two trimesters of pregnancy (IPTp) appears to still provide some benefit, even in the presence of high levels of this common quintuple genotype [9]. However, the WHO recommends surveillance for this genotype, and replacement of SP for IPTp when these quintuple mutant parasites reach prevalence greater than 50%. More recently, in East and Central Africa, parasites with a triple mutant *pfdhps* allele 437**G**/540**E**/581**G** have been observed to abrogate the benefits of IPTp with SP in some areas of East and Central Africa [23, 24].

The **IRN** triple mutant allele of *pfdhfr* was present in 57% of the clinical samples. The triple mutant *pfdhfr* is common in West Africa as reported for neighbouring Benin and Burkina Faso and Gabon in Central Africa [25–27]. In most of West Africa and likely reflects spreading of parasites that carry the imported *pfdhfr* Southeast Asian allele from East African sources [28–30]. The clinical ramifications of the *pfdhfr* triple mutant allele may be largely counterbalanced by the absence of *pfdhfr* I164**L**.

The parasite genotypes of *pfdhps* in Gabi were more complex. The **IRN**/**G**AA haplotype, the most frequent in Gabi, is a marker of a lineage phylogenetically different from those previously identified in Asia and India [31]. Twenty-two % (23/108) of the clinical samples carried a mutation both at codon 437**G** and 613**S**; moreover, nine of these clinical samples carry a triple mutant allele of *pfdhp*s that also include 581**G** (**G**K**GS**). Only one parasite (1/36) observed in Gabi, carried the triple *pfdhfr* with a unique haplotype of *pfdhps,* 437**G**/581**G**. It is not clear whether this strong effect of parasites carrying the 581**G** allele in the absence of 540**E** would be equally deleterious.

The mutations in position 613 of the *pfdhps* gene, and the replacement of an isoleucine residue at position 431 with a valine have seldom been observed outside Niger. The I431**V** mutation was reported the first time in 2007 in Nigeria for a limited number of clinical samples but, in contrast to what was found in Nigeria, the presence of the I431**V** mutation was not associated in Niger with the A581**G** and A613**S** mutations. Instead, it was associated with the S436**A** and A437**G** mutations [26, 32].

These reported prevalences of parasites with genotypes known to compromise SP efficacy in Gabi are, in any case, low and unlikely to generate a significant effect on the clinical outcome following preventive treatment with SP. The distribution and prevalence of these mutations should nevertheless carefully be explored to determine their significance in the response of parasites to SP. Most important, parasites carrying the I164**L** mutation of *pfdhfr* and the K540**E** mutation of *pfdhps* in addition to the “usual” quintuple are even more strongly associated clinical treatment failure and no parasites with this genotype were detected in Gabi before the deployment of SMC with SP + AQ.

Molecular characterization of the *pfcrt* and *pfmdr1* markers facilitates prediction of the responses of parasites to chloroquine (CQ) and AQ, but also to mefloquine and lumefantrine. It has been shown that parasites combining both the *pfcrt* 76**T** and *pfmdr1* 86**Y** mutations and those carrying the *pfmdr1* 86**Y**/184**Y/** 1246**Y** triple-mutant haplotype are resistant to 4-aminoquinolines and associated with clinical failure with AQ treatment [22, 23]. Selection of the *pfmdr1* N86/F184/D1246 haplotype has been reported during treatment with artemether–lumefantrine and in infections with increased sensitivity to mefloquine and decreased susceptibility to lumefantrine [23]. Parasites that carry 2 or more copies of the wild type allele of *pfmdr1* are also highly resistant to both lumefantrine and mefloquine [33]. In Niger, the F184**Y** mutation was found to be the most frequent, with 37% of sequences displaying this mutation. The 86**Y**, 1042**D** and 1246**Y** mutations were less common, with prevalences of around 0.1. In the study region, almost all (88%) the parasites carried the NFD or N**Y**D haplotype, reflecting the selection exerted by AL, the first line treatment in Niger. Parasites carrying the YYY form were not detected. The *pfcrt* sequences were not determined in this study, but other analyses performed in southern Niger during the same period showed that the wild-type CVMNK allele (amino acids 72–76) of the *pfcrt* gene was present in 85% of clinical samples [34]. This is consistent with previous in vitro results, which indicated that the clinical samples from Niger respond adequately to other 4-aminoquinolines like AQ [30]. The *pfmdr1* sequences characterized predict acceptable susceptibility of infections to AQ. Only 11% of the clinical samples carried the *pfmdr1* 86**Y** mutation and the 86**Y**/184**Y**/1246**Y** triple mutant allele was absent. It is also important to recognize that blood-stage resistance mechanisms may not prevent anti-folates from working to some degree in the liver-stages.

In contrast to other countries, SP treatment has never been recommended as a first-line anti-malarial treatment in Niger. The artemether–lumefantrine combination was introduced in 2005, to replace CQ, whereas the use of AQ has remained marginal. The artesunate–amodiaquine combination was introduced later on in 2008 to expand the options for artemisinin-based combination therapy (ACT) available in Niger. The resistance profiles currently observed thus reflect the treatment policies implemented by the health authorities in Niger over the last 10 years. In contrast to this history, the K540**E** mutation of *pfdhps* is found principally in the regions in which SP was used as a first-line treatment to replace CQ before the introduction of ACT. In West African countries in which CQ was replaced directly with ACTs, the K540**E** mutation emerged later. Thus, the strength and order of the drug pressures exerted on the parasite are likely to constrain and structure the parasite populations in a persistent manner [10, 12].

It is important to highlight several key limitations of our study. First, the sample size is both limited and restricted to children presenting at a health centre with fever, so the results presented here may not reflect the prevalence of these genotypes in the general parasite population. However, as children under 5 account for 20% of the total population of Niger, the expected benefit of SMC could be considerable. Although a high prevalence of the triple *pfdhfr* haplotype was observed, the codon 540 of the DHPS gene remained wild type, and SP was likely efficacious at least in 2012. Based on the continued efficacy of SP for prophylaxis in pregnant women, it is possible that even in the presence of the “usual” quintuple (**IRN/GE**A), SP might still retain efficacy for SMC.

Molecular surveys of the prevalence of parasite genotypes can provide useful information to guide decisions on local drug use, particularly for malaria prevention. It would be even more useful if the marker prevalence could be assumed to apply to other locations, or at least sites very close to the site sampled. However, a related study of both SP and AQ molecular markers was completed in 2013 only 15 km away from Gabi, and the prevalence of the *pfdhps* haplotype, 437**G**/540**E** was about 20% at the beginning of SMC implementation, and rose to about 50% by the end of the intervention [34]. Although these samples were collected 1 year apart, this molecular heterogeneity in clinical samples from geographically close sites is not uncommon [28, 35].

This spatial heterogeneity is likely to be common, and presents a challenge for policy makers who have responsibility for setting drug use policies on a national scale for both SMC and IPTp. In any case, regular monitoring of anti-malarial drug resistance should remain a key activity in the sites where SMC and SP IPTp are implemented. Given the sometimes large differences between maker prevalence, even between nearby sites, determination of the molecular marker prevalences in the actual study site should be a high priority to monitor anti-malarial resistance.
